# Integrative Modeling of Gene Expression and Metabolic Networks of *Arabidopsis* Embryos for Identification of Seed Oil Causal Genes

**DOI:** 10.3389/fpls.2021.642938

**Published:** 2021-04-06

**Authors:** Mathieu Cloutier, Daoquan Xiang, Peng Gao, Leon V. Kochian, Jitao Zou, Raju Datla, Edwin Wang

**Affiliations:** ^1^Laboratory of Bioinformatics and Systems Biology, National Research Council Canada, Montreal, QC, Canada; ^2^Aquatic and Crop Resource Development, National Research Council Canada, Saskatoon, SK, Canada; ^3^Global Institute for Food Security, University of Saskatchewan, Saskatoon, SK, Canada; ^4^Centre for Health Genomics and Informatics, Cumming School of Medicine, University of Calgary, Calgary, AB, Canada; ^5^Department of Biochemistry and Molecular Biology, Cumming School of Medicine, University of Calgary, Calgary, AB, Canada

**Keywords:** fatty acids, plant embryo, dynamic modeling, gene expression, metabolic networks

## Abstract

Fatty acids in crop seeds are a major source for both vegetable oils and industrial applications. Genetic improvement of fatty acid composition and oil content is critical to meet the current and future demands of plant-based renewable seed oils. Addressing this challenge can be approached by network modeling to capture key contributors of seed metabolism and to identify underpinning genetic targets for engineering the traits associated with seed oil composition and content. Here, we present a dynamic model, using an Ordinary Differential Equations model and integrated time-course gene expression data, to describe metabolic networks during *Arabidopsis thaliana* seed development. Through *in silico* perturbation of genes, targets were predicted in seed oil traits. Validation and supporting evidence were obtained for several of these predictions using published reports in the scientific literature. Furthermore, we investigated two predicted targets using omics datasets for both gene expression and metabolites from the seed embryo, and demonstrated the applicability of this network-based model. This work highlights that integration of dynamic gene expression atlases generates informative models which can be explored to dissect metabolic pathways and lead to the identification of causal genes associated with seed oil traits.

## Introduction

Fatty acids (FAs) in crop seeds are a major source for human nutrition and potential biodiesel fuels (Tang et al., 2015; Kumar et al., 2016). During seed development, various lipid compounds are synthesized and finally stored in the embryo as energy and nutritional reserves (Wang et al., 2007; Acket et al., 2020). Traditionally, to understand the biochemical processes associated with fatty acid synthesis and metabolism in seeds, single pathway-based approaches have been employed. However, recent evidence presented in multiple studies suggests that the amounts of the storage compounds in plant seeds are affected by multiple interacting pathways, and their associated metabolic networks (Baud et al., 2008; Xu et al., 2014; Allen et al., 2015; Omranian et al., 2015; Ravikrishnan et al., 2018). Therefore, it is important to apply multi-disciplinary approaches including metabolic network engineering to improve the FA contents and their quality in crop seeds.

To guide metabolic engineering, it is critical to understand and develop analytical tools for the complex metabolic systems that operate in seeds (Koch et al., 2017). Metabolic Flux Analysis (MFA) has been used in the developing embryo (Schwender et al., 2003, 2004; Schwender, 2008; Kruger and Ratcliffe, 2012) to quantify metabolic fluxes in the major pathways of carbon metabolism, and these studies showed the contributions of different pathways (e.g., glycolysis and Rubisco C fixation) in producing the building blocs for FA synthesis (Schwender et al., 2004). Metabolic Control Analysis (MCA) (Kacser and Burns, 1973; Moreno-Sánchez et al., 2008), which is used to quantify the amount of control a specific step exerts on a pathway, has shown that the control of oil accumulation in seeds occurs both through FA synthesis (e.g., the FA synthase complex) and through triacylglyceride (TAG) assembly (Ramli et al., 2002). Combined network analysis for prediction of metabolic pathways based on metabolomics data, *in silico* analysis and machine learning was recently conducted in tomato, displaying the potential of artificial intelligence in model simulation of metabolic networks (Beckers et al., 2016; de Luis Balaguer and Sozzani, 2017; Toubiana et al., 2019). However, these modeling efforts are either limited to steady-state conditions or conducted in the context of a single pathway. Thus far, dynamic modeling (i.e., by integrating gene expression data into the modeling) of seed metabolic networks has not been explored in-depth or in great detail.

Dynamic expression of genes and metabolic enzymes in seed development determines seed FA contents (Xu et al., 2014; Acket et al., 2020). In fact, significant phenotypic variation has been observed in seed FA contents (Hobbs et al., 2004; Wang et al., 2007) with, for example, FA content varying from 20 to 45% of the seed weight in *Arabidopsis*. Although it is feasible to obtain gene expression profiles in seeds (Baud and Lepiniec, 2009; Xiang et al., 2011; Gao et al., 2019), their dynamic and quantitative effects on seed FA content is not well defined, and it is thus extremely challenging to identify key genes that accurately determine seed FA content directly from gene expression data. Therefore, to better understand the seed metabolic networks and develop more realistic predictive models for guiding metabolic engineering, it is necessary to model dynamic seed metabolic networks by integrating gene expression profiles (for genes encoding enzymes involved in FA metabolism) during embryo and seed development.

In this study, we present a mathematical modeling approach that integrates the dynamics of gene expression into an Ordinary Differential Equations (ODE) model of the metabolic networks representing the major seed development biochemical pathways in the embryo. Toward this end, we first calibrated the model using the quantitative profiles of the major seed storage compounds (i.e., FA, proteins, and starch) from global metabolomics and gene expression profiles of *Arabidopsis* embryo and seed development. We then systemically perturbed the key network genes to predict the FA contents in *Arabidopsis* seeds. Finally, the predictions have been validated by published work and new experimental data.

## Materials and Methods

### Plant Materials and Growth Conditions

*Arabidopsis thaliana* wild type (*Col-0*) and mutants (*acc –* SALK_017342 and *epi –* SALK_145945) were grown under 16 h light/8 h dark photoperiod with constant temperature of 22°C at 120 μE m^–2^ s^–1^ light intensity. The insertion positions of SALK T-DNA lines were confirmed by PCR using the following primers. *acc* : LP-TTCAAGCAAGTTCAGGGTGAG, RP-AGAAGTACGCCCACACATTTG; *epi* : LP-GTTCATCAA CCCAGGTCAATG, RP-CCTTCTCTGCACACATTTTCC.

### Embryo Dissection and Microarray Experiments

Embryo isolations, RNA extractions, microarray experiments, microarray normalization and bioinformatics analysis were performed as described previously (Xiang et al., 2011). In this study, seven stages of *Arabidopsis* embryo were isolated, including zygote, octant, globular, heart, torpedo, bent, and mature stages. The mean gene expression matrix for the microarray data was based on our previous published report presented in Supplementary Table 1 of Xiang et al. (2011), Plant Physiology.

### Metabolite Profiling of Embryos

The embryo samples from the same seven stages as the samples used for microarray analysis in Xiang et al. (2011) were isolated for metabolic analysis. Dissected embryos of the same stage were pooled in tubes on dry ice and kept in −80 refrigerators. Tissue samples were ground in liquid nitrogen and freeze dried for 6 days under vacuum. Four biological replicates for each of these samples were further processed and analyzed by Metabolon (Morrisville, NC, United States) for global unbiased metabolic profiling involving a combination of three platforms: ultra-HPLC (UHPLC)-tandem mass spectrometry (MS/MS) optimized for basic species, UHPLC/MS/MS optimized for acidic species, and gas chromatography-MS. The methods used were the same as described previously (Evans et al., 2009; Oliver et al., 2011). For *acc* and *epi* mutants, there is no significant difference between the two mutants and wildtype plants before the heart stage. The seeds of homozygous *acc* mutants turn white and become smaller than wildtype after the heart embryo stage, and the seeds of homozygous *epi* mutants turn white and become smaller than wildtype after the bent embryo stage. Thus, we used the samples of the two mutants at the heart stage for metabolite profiling. Fatty acid content was calculated based on all compounds in the fatty acid sub-pathway. The fatty acid content of the samples from the two mutants and wildtype at the heart stage were compared.

### Mathematical Treatment of Gene Expression Profiles

**FIGURE 1:**
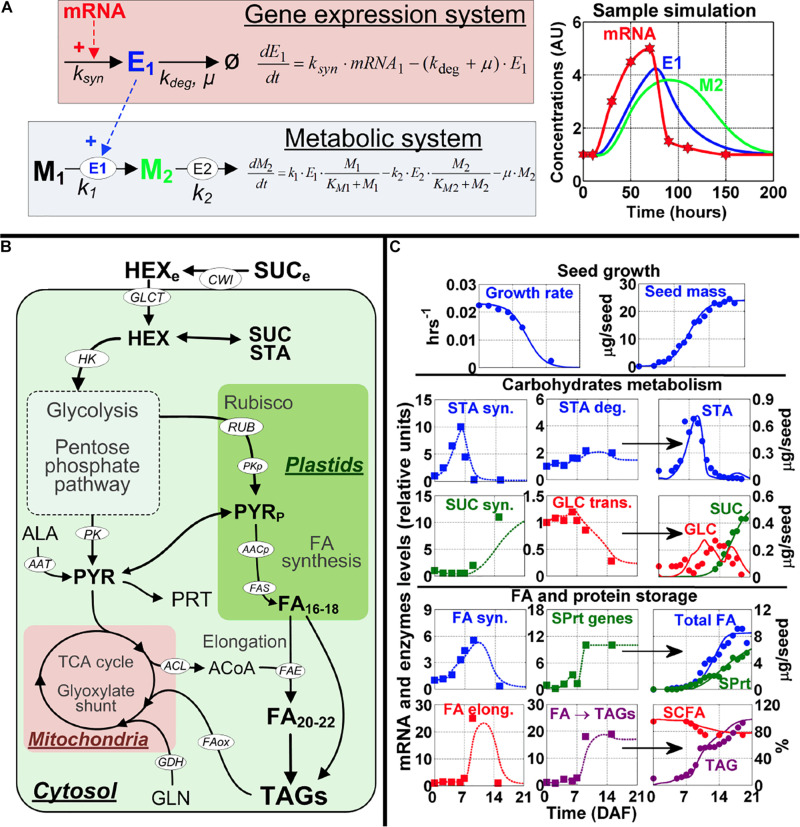
Modeling framework and model calibration. **(A)** The modeling approach to map gene expression dynamics onto the metabolic network. The kinetic equations (left part of **A**) for modeling the gene expression system and metabolic system were developed based on data for mRNA profiles from microarray and metabolite content from global metabolic analysis for the seven embryo developmental stages. mRNA (red) modulates enzyme synthesis (E1, blue) which in turn catalyzes the production of a product metabolite (M2, green) from a substrate metabolite (M1, black). A sample simulation is shown with a transient mRNA profile (red stars) used as an input (right part of **A**). The symbol legend for the kinetic equations: *k*, enzyme reaction constant; *k*_syn_, the rate constant for enzyme synthesis (hr^− 1^); *k*_deg_, the rate constant for enzyme degradation (hr^− 1^). μ, the growth rate of the cell (hr^− 1^). **(B)** Overview of the metabolic pathways important for *Arabidopsis* embryo physiological development; a complete set of differential equations and further details for this model are presented in Supplementary Figure 2. Substrates, metabolites and end products are in bold font, cofactors are in gray font, and enzymes and reactions are in white ellipses. Full names of enzymes and metabolites are provided in the Supplementary Tables 3–12. **(C)** Model calibration using wild-type *Arabidopsis* physiological data, including seed growth (top row of graphs), carbohydrate metabolism (middle rows of graphs), and FA and storage protein accumulation (bottom rows of graphs). In each row graphs presenting data on carbohydrate metabolism and FA and storage protein accumulation, the first and second columns of graphs depict mRNA data from the seven embryo developmental stages (squares) and simulated enzymes levels (dotted lines), which are used for simulation of content for starch (STA), sucrose (SUC), glucose (GLU), total fatty acids (total FA), storage proteins (SPrt), short-chain fatty acids (SCFA) and triacylglycerides (TAG). The simulation results (solid lines) are shown in the third column. Circles in the simulation graphs in the 3rd column of graphs are experimental data from Baud et al. (2002) to show the simulations are close to experimentally derived results. Key for other abbreviations used in **(C)**: STA syn., genes and enzymes involved in starch synthesis; STA deg., genes and enzymes involved in starch degradation; SUC syn., genes and enzymes involved in sucrose synthesis; GLC trans., genes and enzymes involved in glucose transport; FA syn., genes and enzymes involved in fatty acid synthesis; SPrt genes, genes and enzymes involved in storage protein synthesis; FA elong., genes and enzymes involved in fatty acid elongation; and FA - TAGs, genes and enzymes involved in triacylglyceride formation.

Some hypotheses and simplifications were necessary in order to integrate the gene expression profiles in the plant embryo metabolic network (c.f. Figure 1A). First, relative mRNA and enzyme levels (i.e., scaled around a value of 1 for the first data point) were used in order to circumvent the absence of correlation between mRNA and protein concentration across the genome (Greenbaum et al., 2003). An integrated study of 319 transcript/protein pairs in *Arabidopsis* seeds revealed a poor correlation as well (Hajduch et al., 2010). It is thus important to highlight that our modeling framework does not imply a direct correlation between mRNA and protein (i.e., because we consider the dynamics). In the published dataset previously mentioned (Hajduch et al., 2010), we identified 16 genes that are present in our model and we observe that post-translational modifications are not significant for 72% of the genes in our model. This is higher than the average of 56% for the *Arabidopsis* genome (Hajduch et al., 2010). However, it is clear that the consideration of posttranslational modifications could be a further improvement to the model when sufficient quantitative information is available. Regarding the dynamics of the gene expression model, the turnover rates for the enzymes are simplified into three groups (fast, average and slow turnover rates), based on literature data, when available (Piques et al., 2009).

### Model Implementation and Calibration

The model was implemented using the Systems Biology toolbox (Schmidt and Jirstrand, 2006) for Matlab (The Mathworks Inc., Natick, MA, United States). The ODE’s and kinetic equations for fluxes were implemented in the toolbox with first estimates for parameters taken from an extensive review of enzyme kinetics literature^1^ (Chang et al., 2009). The Michaelis–Menten equation was used to describe enzyme activity with regard to substrate concentrations and the Hill equation was used to describe biochemical feedback inhibition. Modeling approaches such as the log-linear approximation were used to reduce the number of parameters in large models (Visser et al., 2004). However, this method involves the linearization of the kinetics around a certain reference point, usually at steady-state. This is not automatically applicable in seeds because the large changes in seed gene expression can induce large changes at the metabolic level (i.e., some enzymes, substrate concentrations or fluxes can vary 5-10-fold).

The calibration of the model’s parameters was done through an iterative process consisting of three major steps:

(1) A round of rough tuning of the parameters to produce a numerically stable and realistic model, with concentrations and fluxes within physiological ranges from reported datasets (Schwender et al., 2003, 2004; Schwender, 2008).
(2) The parameter estimation routines in the SBtoolbox are then used to optimize the parameters of the model in order to fit quantitative experimental data (Baud et al., 2002).
(3) If step #2 cannot yield satisfactory results, we consider changing the structure of the model either by implementing metabolic reactions or feedback regulation mechanisms that were previously not included. New regulatory mechanisms are always verified against the most recent literature and databases (Chang et al., 2009).

These steps were repeated multiple times and the final result was a robust model that can be simulated over the complete time course of development (0–21 days after fertilization, DAF) and very good agreement was achieved with regard to available data. The following is a complete description of the ordinary differential equations (ODE) model, which includes the differential equations and kinetic equations for fluxes and parameters, with further discussion on parameter estimation and the problem of model under-determination.

### Complete Description of the ODE Model for *Arabidopsis thaliana* Seed Dynamic Metabolic Network

#### Gene Expression Model

The model for gene expression consists of one differential equation per enzyme, in the following form:

(1) $$\frac{dE_{\text{i}}}{dt}=k_{\mathrm{syn},i}\cdot mRNA_{\text{i}}\cdot Rb-\left(k_{\deg,i}+\mu \right)\cdot E_{\text{i}}$$

Where *E*_*i*_ is the amount of the ith enzyme in the pathways, for which the synthesis and degradation are determined by the following parameters:

• mRNA_*i*_: the amount of mRNA that encodes for the enzyme (dimensionless);
• Rb: the amount of ribosomes in the cell (dimensionless);
• *k*_*syn,i*_: the rate constant for enzyme synthesis (hr^–1^);
• *k*_*deg,i*_: the rate constant for enzyme degradation (hr^–1^), which = LN(2)/*t*_1/2_;
• μ: the growth rate of the cell (hr^–1^).

Thus, the enzyme synthesis rate (first term on the right hand side) is proportional to the amount of *mRNA*, *Rb* and the rate constant for synthesis (*k*_syn_). The enzyme concentration can be reduced by two phenomena. First, the enzyme can be degraded (*k_deg_⋅E*) or the enzyme pool can be diluted by cell division (*μ⋅E*). Note that the protein degradation rate constant is inversely proportional to protein half-life (*t*_1/2_). Thus, the problem here is to determine realistic parameters so that we can solve the differential equation for each *E*_*i*_. The following subsections will detail the hypotheses and development of this gene expression model.

##### Units

In this framework, the parameter’s time units will be given in hours. Given that the enzyme levels are not used in absolute terms for direct comparison with experimental data, it was decided to use dimensionless (i.e., relative) concentrations for *mRNA*, *E* and *Rb*. Justification for this approach is given below. Also, the model implementation was performed so as to circumvent the use of relative concentrations for these variables. Specifically, the enzyme level (*E*_*i*_) is the only variable to be connected to the rest of the metabolic network (i.e., it increases the maximal rate of its reaction) and we will scale the enzyme levels by using a rate constant (*k*_*cat*_). For example, the maximal reaction rate of the ith reaction in our metabolic network is:

(2) $$V_{\max,i}=E_{i}\cdot K_{i}$$

In that equation, the maximal enzyme activity (*V*_*max,i*_) will be proportional to the enzyme level (*E*_*i*_) and enzyme reaction constant (*k*_*i*_). As we have to determine parameters for each reaction (i.e., the *k*_*i*_), the absolute value for *E*_*i*_ is not critical because it is multiplied by *k*_*i*_. Thus, finding a value for *k*_*i*_ (with a relative amount of *E*_*i*_) or finding a value for both *k*_*i*_ and *E*_*i*_ is the same problem in terms of curve fitting (i.e., considering absolute concentrations for *E*_*i*_ cannot improve the fit). In terms of model development, we thus ‘lump’ the absolute enzyme level in the parameter *k*_*i*_. What is important, though, is that if the mRNA level doubles (for example), we would like to have twice the enzyme concentration and twice the maximal reaction rate for the corresponding reaction (after a steady-state is reached). In that regard, equations S1 and S2 will achieve this.

##### mRNA levels (mRNA_*i*_)

mRNA levels were taken from our gene expression map of *Arabidopsis* embryo development^2^ (Xiang et al., 2011). The data set for mRNA covers the whole development of the seed, with early stages included. Supplementary Table 1 presents the seed developmental stages for which mRNA data was acquired.

Most enzymes in metabolism are encoded by more than one gene and some enzymes are localized in more than one cellular compartment (i.e., cytosol, mitochondria, and plastids). To classify the enzymes in the model, we used the information available in KEGG (Kyoto Encyclopedia of Genes and Genomes^3^) and BRENDA^4^ (Chang et al., 2009). Thus, for each enzyme, we sum the mRNA of genes that are expressed (see details below).

One important point to consider here is that there is no absolute and quantitative correlation between mRNA levels and protein concentrations across the genome (Greenbaum et al., 2003). Protein A might be present in higher concentration than protein B, even though, for example, mRNA_*B*_ > mRNA_*A*_. We thus circumvent this problem by using normalized (i.e., dimensionless) amounts. We will thus normalize all of our mRNA profiles with regard to the initial value (i.e., mRNA at stage Z) in order to scale all the initial mRNA levels to around 1:

(3) RmNAi(t)=∑jmRNAi,j(t)∑jmRNAi,j(t=0)with'j′=numberofgenesforenzyme'i′

where mRNA_*i*_(t) is the normalized sum of mRNAs for enzyme ‘*i*’ at time ‘*t*’ in the metabolic network.

##### Ribosome (Rb)

Genes that encode for ribosomal subunits are known for *Arabidopsis* (Barakat, 2001) and we identified these (*n* ≈ 200) in our gene expression database. Among the 200 genes, ≈150 were expressed at an average intensity of 2,000 or more and ≈100 were highly expressed (intensity > 10,000). Since little information is available on the half-life of ribosomal proteins in *Arabidopsis*, we simply assumed that the amount of ribosome is proportional to the weighted sum of mRNAs for the ribosomal genes (mRNA_*Rb*_). Research with *Chlamydomonas reinhardtii* suggests such a correlation exists (Martin et al., 1976). As for the other mRNAs, the scaling was done so as to have Rb(*t* = 0) = 1.

##### Growth rate (μ) and enzyme degradation (*k*_deg_) and synthesis (*k*_syn_)

In many circumstances, the dilution by growth (*μ⋅E*) can be disregarded, especially in non-dividing cells and tissues or for enzymes that have a very high turnover rate (i.e., *k*_deg_ >> μ). As it is, the case of plant embryos is an interesting situation where the growth rate and enzyme degradation are on a similar timescale and we must thus consider both phenomena.

Growth rate (μ) was estimated from published data (Baud et al., 2002), with an average rate of 0.015 hr^–1^ (doubling time of ≈48 h). A similar value of 0.014 hr^–1^ was also reported in research on plant metabolic flux analysis (Schwender et al., 2003). The growth rate will, however, change during embryo development, with a value of 0.025 hr^–1^ in the early stages and a much lower growth rate (0.0025 hr^–1^) for the mature embryo. In the modeling, this is reproduced by using a logistic growth equation (Figure 1).

Measurement of overall protein turnover in seeds by labeling techniques (Holleman and Key, 1967) yielded an average protein turnover rate (*k*_deg_) of 0.025 h^–1^, which is on the same order of magnitude as the growth rate. This is much faster than turnover rates in other plant tissues, as values in the range 0.001–0.005 hr^–1^ are reported for leaves (Huffaker and Peterson, 1974), with the suggestion that a correlation exists between protein turnover and tissue growth. Specific values of *k*_deg_ for enzymes in *Arabidopsis* metabolism are also reported (Piques et al., 2009), but significant variability is mentioned as a major problem in estimating turnover rates for each protein. Given this variability, and in order to reduce the number of parameters, we will only consider three ‘groups’ for the turnover rates of enzymes in our model:

(1) Enzymes with a relatively fast turnover rate (*k*_deg_ = 0.03 hr^–1^).
(2) Enzymes with an average turnover rate (*k*_deg_ = 0.02 hr^–1^).
(3) Enzymes with a slow turnover rate (*k*_deg_ = 0.01 hr^–1^).

This simplification is of course arbitrary, but it does reduce the number of parameters in the model, while keeping the turnover rates of enzymes in physiological ranges. Enzymes in our model were classified according to reported results (Piques et al., 2009). If no information on a specific enzyme was available, the default option is to use the average turnover rate.

Finally, the rate constant for protein synthesis (*k*_*syn,i*_) needs to be determined. Again, limited information is available in the literature, but we can use the same approach as for protein degradation, with a classification in three groups (low, average, and fast turnover). Data from an integrative study (Baud et al., 2002) show that, on a mass basis (i.e., gr. protein per gr. seeds), the amount of protein (i.e., non-storage protein) does not change during most of the embryo development. This implies that protein production and removal (because of growth and degradation) are mostly at equilibrium. As we estimated degradation and growth, we can balance equation S1 so that, initially (i.e., at *t* = 0, when μ = 0.025 hr^–1^), protein synthesis will match the turnover because of growth and degradation (*k*_syn_ = *k*_deg_ + *μ*). This gives us the following parameters for the three groups:

(1) Enzymes with a relatively fast turnover rate (*k*_syn_ = 0.055 hr^–1^)
(2) Enzymes with an average turnover rate (*k*_syn_ = 0.045 hr^–1^)
(3) Enzymes with a slow turnover rate (*k*_syn_ = 0.035 hr^–1^)

Thus, even though this modeling of enzyme level is based on some simplifying assumptions and scaling to have dimensionless enzyme levels, it does have significant advantages, namely: (a) limited number of parameters (*k*_syn_ and *k*_deg_); (b) mRNA, Rb and μ are based on reliable data; and (c) the dynamics of gene expression are described in a physiologically realistic framework.

This framework for the modeling of enzyme levels will thus be integrated (c.f. Figure 1A) in a metabolic model for the major pathways of carbon storage and energy metabolism in the developing *Arabidopsis* embryo. The metabolic layer of this model is presented in the manuscript and detailed equations are given in Section “Metabolic Model” below.

##### Data and simulation for mRNA and enzyme levels

As detailed previously, the mRNA data was acquired at 7 stages during embryo/seed development (Xiang et al., 2011). Supplementary Table 1 gives a list of these developmental stages. Supplementary Table 2 gives a list of the genes used for each enzyme of the model, with its corresponding turnover rate (i.e., fast, average, or low). Supplementary Figure 1 presents normalized time profiles for mRNAs (i.e., equation S3) and corresponding enzyme dynamic profiles (i.e., equation S1 solved for each enzyme).

##### Transcript/enzyme and posttranslational modifications

Finally, it can be argued that even for a single transcript/protein combination, a correlation is not fully guaranteed, mostly because of posttranslational modifications. A study of 319 transcript/protein pairs in *Arabidopsis* showed that the correlation is observed only in 56% of the cases (Hajduch et al., 2010). However, 16 genes from our model are present in this dataset and among these, 12 had a good correlation for the slope and 11 for the curvature [as defined in Hajduch et al. (2010)], for an overall agreement of 72% (23/32). We thus assume that not considering posttranslational modifications is a reasonable hypothesis at this point, especially as little information is available to correctly implement this phenomenon.

Moreover, and as discussed in the manuscript, our modeling framework does not assume a direct correlation between transcript and enzyme, mostly because we consider the dynamics of the system and changes in ribosome amount. As can be seen from Supplementary Figure 1, the correlation between mRNA/enzyme is not direct in many cases, especially for enzymes with slow turnover rates, such as Rubisco (RUB).

#### Metabolic Model

This section presents the metabolic model as a set of ODE’s obtained from the mass balances for metabolites. The dynamics of the system are then described using kinetic equations for the regulation of fluxes. For clarity, the metabolic system will be divided into the following subsystems, which will be presented separately:

• Central metabolic pathways (glycolysis, pentose phosphate etc.) in Supplementary Tables 3, 4.
• Plastid metabolism and FA pathways in Supplementary Tables 5, 6.
• Mitochondrial metabolism in Supplementary Tables 7, 8.
• Pathways for growth, storage product accumulation and transport in Supplementary Tables 9, 10.
• Kinetic equations for maintenance fluxes and FA oxidation in Supplementary Table 11.
• Parameters of the model in Supplementary Table 12.

Then, a detailed diagram of the metabolic pathways is presented in Supplementary Figure 2.

##### Mass balances and kinetic equations

The generic form of a metabolic model is:

(4) $$\frac{dM}{dt}=S\cdot v-\mu \cdot S$$

…where *M* is the vector of metabolites, *S* is the stoichiometric matrix of the system (determined from the topology of Supplementary Figure 2) and μ is the growth rate. *S* is an m-by-n matrix, where ‘*m*’ is the number of reactions and ‘*n*’ is the number of metabolites. Each row of *S* thus indicates which metabolites are taking part in the mth reaction. The rate of change in metabolite is described by the product of the stoichiometry matrix and the flux vector (*v*), as well as by a dilution term (μ*⋅S*) which accounts for the increase in volume because of cellular growth (μ).

In this model, the kinetic equations for flux regulation will have the following form:

(5) $$v_{i}=E_{i}\cdot k_{i}\cdot f\left(M,p\right)$$

…where *E*_*i*_ is the amount of enzyme catalyzing reaction ‘*i*’ (modeled as described in Section “Gene Expression Model” above), *k*_*i*_ is the reaction constant for this enzyme and *f(M,p)* will be a function of the state of the system (*M*) and constant parameters (*p*). This function will use Michaelis–Menten, Hill and mass-action kinetics to account for the various substrates, co-factors and inhibitors involved in reaction ‘*i*’. A higher value for the Hill coefficient for a reaction indicates the sensitivity at low input becomes a threshold response, such that a minimal input is needed to stimulate significant change in output. Such a response seems to reflect realistic biological systems.

##### Central metabolism pathways

The central metabolic pathways in our model include glycolysis and the pentose phosphate pathway (PPP) and these are connected to other pathways. Glycolysis is described in a simplified way, with 5 reactions [hexokinase (HK), phosphofructokinase (PFK), phosphoglycerate kinase (PGK), non-phosphorylating glycerate dehydrogenase (NPG) and pyruvate kinase (PK)]. We included the non-phosphorylating glycerate dehydrogenase (NPG), which operates in parallel to glycolysis and produces NADPH that can be used for FA synthesis. However, this is not the major source of NADPH, as most of it is produced by the PPP. Ribose-5-phosphate (R5P), the intermediate product of the PPP, can link and integrate the pathways through two different mechanisms. The epimerase (EPI) reaction will direct R5P back to glycolytic intermediates, while Rubisco (RUB will generate downstream glycolytic intermediates in the plastids. The final product of glycolysis, pyruvate (PYR), can be used in many pathways. It connects to mitochondrial metabolism, but can also be transported in the plastids for FA synthesis or can be used for amino acid and ultimately, protein synthesis.

The structure of the model, with the seeds operating in glycolytic mode (i.e., we don’t consider gluconeogenesis) is in agreement with what is proposed by seed metabolic flux estimations (Lonien and Schwender, 2009). The model also accounts for the energy (ATP) and redox balance (NADH and NADPH). This limits the range of operation to realistic, physiological conditions by respecting the fundamental thermodynamic limits inherent to the metabolic system.

It is well known that the pathways of central metabolism are regulated at the biochemical level in most organisms, including plants (Plaxton, 1996). We have implemented many of these well known biochemical feedback mechanisms, as can be seen from the various inhibition kinetics implemented in the equations for metabolic fluxes in Supplementary Table 4. Again, all of these mechanisms are verified against available information in public databases^5^.

##### Plastid metabolism and FA pathways

The structure of the model for plastid metabolism is based on measured fluxes (Lonien and Schwender, 2009), with a contribution by Rubisco to provide carbon backbones for FA synthesis. The synthesis, elongation and storage of FA into oil bodies is based on a structure proposed by the analysis of mRNA data to identify active pathways in FA synthesis (Baud and Lepiniec, 2009). We simplified our model to FA’s of lengths between 16 and 22C and did not consider isomers.

##### Mitochondrial metabolism and energy

The structure of our model for mitochondrial metabolism was again chosen to be coherent with flux measurements (Lonien and Schwender, 2009), with the addition of the glyoxylate cycle (simplified to the isocitrate lyase reaction). Connections between the TCA cycle and other pathways are also implemented, with a contribution of phosphoenolpyruvate carboxylase (PEPC), malic enzyme (ME), glutamate dehydrogenase (GDH), alanine aminotransferase (AAT), and ATP citrate lyase (ACL), all of which are reported to be significant for the overall balance of embryo metabolism (Lonien and Schwender, 2009). Insights for the regulation, kinetic equations and concentrations were taken from a generic model of mitochondrial metabolism (Nazaret et al., 2009).

##### Growth and storage products accumulation

Reports on flux estimations (Lonien and Schwender, 2009) show that storage products such as proteins (SPRT), starch (STA) and sucrose (SUC) are important sinks for metabolic fluxes. We thus included these products in our modeling. STA accumulation is simplified to two reactions, one for synthesis (*v*_*stas*_) and one for degradation (*v*_*stad*_). SUC accumulation is driven by the reversible enzyme, sucrose synthase (*v*_*sus*_), and storage proteins are accumulated by a simple reaction whose rate (*v*_*sprt*_) is proportional to mRNAs for storage proteins. Growth is modeled using a logistic equation, with a maximal limit on seed weight. This simplification was implemented for two reasons: it precisely describes the growth curve of *Arabidopsis* seeds and it allows focusing the model on storage product dynamics instead of having to describe detailed mechanisms for growth regulation in seeds (some of which are not clearly defined at the genetic level).

##### Maintenance and FA oxidation

As we model the dynamics of seeds metabolism up to the mature stage, where FA levels are stabilized, we have to consider FA oxidation. We thus implemented reactions for triacylglycerol (TAG) oxidation and subsequent processing of ACoA in the glyoxylate pathway (simplified to the isocitrate lyase reaction).

It is also important to consider reactions for maintenance. The major sources of damage and loss of efficiency in metabolism come from the mitochondrial proton leak and endogenous oxidative stress. The mitochondrial proton leak (*v*_*leak*_) is modeled as a simple reaction that consumes NADH, with estimates based on published modeling (Nazaret et al., 2009). The endogenous oxidative stress (*v*_*ox*_) is modeled in a similar vein, but is based on consumption of NADPH. Finally, we use a mixed model for ATP consumption, with growth associated (α⋅μ) and non-growth associated (β) terms. It is important to highlight that these reactions operate in addition to energy and redox consumption by other pathways in the model (such as FA synthesis) and account for energy consumption by reactions not in the model.

##### Parameter identification

Obviously, this model with 34 metabolic states, 40 reactions (and their associated genes and enzyme dynamics) and around 125 parameters is under-determined and the parameters cannot be uniquely identified. However, the model’s parameters were first taken from published reports (especially the affinity and inhibition constants for enzyme kinetics) when available and the model was fine-tuned using >100 data points (with concentrations and fluxes data) covering the major pathways in the developing embryo. Thus, even though the parameters are not strictly identifiable, it is assumed that the resulting model is within physiological ranges and can produce valuable insights. In a previous metabolic modeling study on plant cells (Cloutier et al., 2009), a similar problem of under-determination did not hinder the analysis and predictive capacity of the model. An even larger model for cell signaling (Chen et al., 2009), with hundreds of states and parameters, was shown to be insightful, as long as it is trained against experimental data, as our model is.

## Results and Discussion

### Constructing and Calibrating the Dynamic Metabolic Network Model

Ordinary Differential Equations (ODE) modeling of metabolic networks is an established framework, with decades of development and applications. Regarding the metabolism of plant cells and tissues, the ODE approach has been applied to photosynthesis (Poolman et al., 2004), sucrose metabolism (Rohwer and Botha, 2001), and metabolism of roots and cell cultures (Cloutier et al., 2007, 2009). Usefulness of such models in predicting results, providing testable hypotheses and improving experimental design for *in vitro* cultures has also been demonstrated (Cloutier et al., 2009).

To build the network model for *Arabidopsis* seeds metabolism, we used the resources and information from KEGG and metabolic flux measurements of *Arabidopsis* seeds (Lonien and Schwender, 2009). This network includes the major pathways for energy production and biosynthesis of storage products in developing embryo, with the major substrates coming from the maternal tissue. The model is also compartmented between cytosol, plastids and mitochondria (Figure 1B). Because the information for these three compartments’ volumes is not available for *Arabidopsis* embryo developmental stages, we assumed the volumes of these three subcellular compartments are equal in this model. Ideally, the model could incorporate the actual volume information to the differential equations in each compartment to adjust concentrations of the metabolites in each compartment. By doing so, we could more accurately model the reactions and make more accurate predictions. This limitation will be addressed in the future when the respective compartment volumes are determined and available for embryo development in *Arabidopsis*. However, it should be noted that even with this limitation regarding model assumptions, as seen in Figure 1 we were able to obtain good agreements between model simulation results and experimental data. This does not mean that the model will not be improved if we provide actual column ratios for the 3 subcellular compartments of cells of the plant embryo, as we expect that will improve the accuracy of the model. A complete picture of the model is provided in Supplementary Figure 2.

Specifically, the model describes glycolysis, mitochondrial metabolism, starch (STA) synthesis and degradation, the pentose-phosphate pathway (PPP), Rubisco (RUB), synthesis of storage proteins (SPRT), and the synthesis, elongation and storage of FA’s into oil bodies (triacylglycerides or TAG). We also include the contribution of the amino acids alanine (ALA) and glutamine (GLN). The topology for the pathways is in accordance with reports on net flux estimations (Schwender et al., 2004; Schwender, 2008; Lonien and Schwender, 2009). We also included reactions for the degradation of TAG and the glyoxylate cycle for FA oxidation. The model also considers the energy and redox balance of each reaction and this constrains the fluxes to realistic, physiological values. Finally, seed growth was modeled using a logistic equation (Figure 1 and Supplementary Table 9).

To integrate gene expression values to the model, we used gene expression profiles of seven distinct embryo developmental stages from zygote to maturity reported in Xiang et al. (2011) study. We considered the dynamic expression changes of the genes to modulate the reactions of the metabolic network. This framework is summarized, conceptually, in Figure 1A. An overview of the model implementation and calibration is provided in the Section “Materials and Methods,” which includes equations, parameters and further references for model development.

To obtain a realistic model, using the gene expression profiles of the embryo development as inputs, we calibrated the model parameters by multiple rounds of validation and curve fitting using the quantitative measurements of the storage products in different developmental stages of wild-type *Arabidopsis* seed (see section “Materials and Methods”). Results obtained with this approach are shown in Figure 1C, where model simulations (full lines) are compared to experimental data (circles).

The simulations for embryo growth rate and total mass are in close agreement with experimental data (Figure 1C, top row). This is important because the rate of cell division in the seed dilutes the enzymes, metabolites and storage products pools (Supplementary Tables 3–11) and this is a major sink for metabolic fluxes (Schwender et al., 2004; Schwender, 2008; Lonien and Schwender, 2009). The dynamics of storage products accumulation are also reproduced with good precision (Figure 1C: STA, SUC, SPRT, FA, and TAG), which suggests that the model accurately integrates the underlying genes’ expression data (Figure 1C, squares). As regards to FA accumulation (Figure 1C, 4th row), the genes that encode for FA synthase are downregulated in the mature stage and this corresponds to stable or slightly decreasing FA levels. Another important observation reproduced by the model is the critical importance of FA elongation and storage in increasing total FA content. Not only does each round of FA elongation add 2-carbon, therefore increasing the length and mass of the fatty acid chain, it has also been established that short-chain fatty acids (SCFA) in the forms of acyl-ACP in the plastids down-regulate the synthesis of FA, by inhibiting the enzymes ACCase and FA synthase (Knoche et al., 1973; Shintani and Ohlrogge, 1995) (Supplementary Table 2). With these mechanisms implemented in the model, we observed that the maximal rate of FA accumulation occurs between 7 and 14 DAF, which corresponds to the only period where FA synthase, FA elongation and TAGs genes are expressed (Figure 1C, 4–5th rows). Total FA concentration is stable in the mature stage (16–19 DAF). Estimation from model simulations revealed the FA turnover rate at 0.06 d^–1^ for this period. This is ≈5 times higher than values reported for leaves (Yang and Ohlrogge, 2009), but this is perfectly consistent with other measures, such as protein turnover, which is also 5 times faster in seeds compared to leaves (Huffaker and Peterson, 1974).

### Simulation for Physiological Fluxes and Their Ratios

Many physiological ratios are measured and considered as insightful in tracking events in embryo development. For example, the HEX to SUC ratio is important in the transition from storage of carbohydrates to embryo dormancy and this ratio was measured experimentally, with a clear peak around 7–10 DAF (Baud et al., 2002). Given that the model simulates the most important cellular states and molecules, we have used the simulation results to calculate these physiological ratios. Supplementary Figure 3 presents a sample of such calculations.

The ratios calculated from model simulations are in close agreement, at least qualitatively, with many experimental reports. Our calculation for the HEX/SUC ratio is similar to what has been reported (Baud et al., 2002). The ratio of PK flux between cytosol and plastids, as well as the contribution of Rubisco to FA biosynthesis (*v*_*RUB*_/*v*_*FAB*_) are consistent with fluxes measurements (Schwender et al., 2003; Lonien and Schwender, 2009), when it is observed that the PK flux is higher in the plastids and that there is a non-negligible contribution of RUB to FA synthesis. The ratio of ATP/ADP, showing an increase during the transition to mature embryo, is also consistent with an experimental report (Borisjuk et al., 2004).

We considered these measurements in developing our model and the simulations. In general, the results presented in Figure 1C and Supplementary Figure 2 show that the model reproduces the precise timing of metabolic events in seeds. These results suggest that the dynamic model is able to reproduce the dynamics of the storage products in *Arabidopsis* seeds.

### Systematic *in silico* Analysis of FA Content After Gene Perturbation and Experimental Validation

**FIGURE 2:**
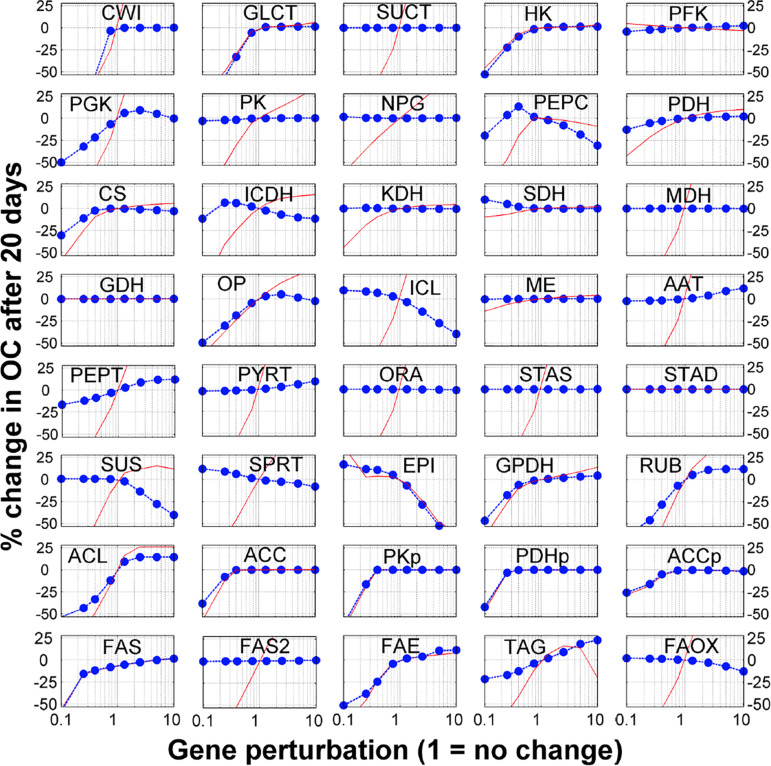
Sensitivity analysis of fatty acid to gene modulation. Each reaction in the model was perturbed by changing the gene expression (horizontal axis) and the *y*-axis shows the % change in fatty acid after 20 days (blue dots). The red lines show the % change in the corresponding metabolic flux between 10 and 20 DAF (i.e., when FA are accumulated).

The model contains 40 reactions. We sequentially perturbed (i.e., gene knockout, by reducing the gene expression values or gene overexpression, by increasing the gene expression values) each reaction and used the model to generate the simulated seed FA content at the mature stage (at 20 DAF). This allows simulating the sensitivity of seed FA contents to genetic perturbations. Complete and detailed results for single gene perturbations, with consideration for 1. 5-, 2. 5-, 5-, and 10-fold increases and decreases are presented in Figure 2. Interestingly, these simulations reproduce the recognized problem of ‘asymmetry’ in increasing the flux in a pathway, whereas it is much easier to reduce the flux in a pathway than to increase it (Fell, 1998; Morandini, 2009). Interestingly, 15 of the 40 genes’ modulation profiles shown in Figure 2 have this ‘asymmetrical’ trend where an increase in gene expression leads to moderate or no increase in FA content, while a decrease in gene expression leads to a significant (and often linear) decrease in FA content. On average, the single gene knock-out (KO) or overexpression (OE) leads to 10 and 0.1% decrease in FA content, respectively. These results suggest that the possibilities offered by modulating candidate genes for metabolic engineering should be evaluated carefully.

Finally, the sensitivity analysis results show that genes related to transport processes might be important for FA synthesis. The breakdown of external sucrose by the sucrose invertase (CWI) and the glucose transporter (GLCT) are two genes for which a KO induces the sharpest decrease in FA content. Other transport associated processes also show some potential to actually increase FA content. Overexpression of alanine aminotransferase (AAT), which incorporates amino acids from the maternal tissue, increases FA content by 8%. Transporters for PEP and PYR on the plastidial membrane (PEPT and PYRT in Figure 2) could both increase FA content (9 and 7%, respectively). As these transporters favor the flow of carbon from the cytosol to the plastids, their overexpression changes the balance of carbon flow toward FA synthesis in the plastids. This observation on the sensitivity of FA content accumulation to transport processes is consistent and in agreement with Metabolic Control Analysis of starch accumulation in potatoes (Morandini, 2009).

To validate these predictions, we searched relevant published work and found that 10 genes in the model have been explored using knockout or overexpression in *Arabidopsis* to measure their FA contents. Interestingly, the experimental results from these studies are in agreement with the simulated results (Table 1). For example, the model predicted that down-regulation of the genes in glycolysis or plastidial pyruvate kinase leads to low FA content in the seed. These cases have been reported previously (Table 1). Moreover, less intuitive results are also reproduced. For example, our model predicted that overexpression of genes for components of the FA synthase complex does not improve FA content. Interestingly, such cases have been reported in the literature (Table 1). At the enzyme level, some of these steps are known to be inhibited by their own products (Supplementary Table 6), and an increase in enzyme amount will not necessarily lead to significant increases in flux because of an immediate negative feedback. We further predicted that modulations for FA elongation or TAG formation result in significant increases in FA content (+10–40%), which are also supported by published work (Table 1). We realized that the key element of making correct predictions is that the model considers the inhibition of the FA synthase reaction by SCFA (Knoche et al., 1973). Indeed, the mechanism of direct feedback inhibition of plastidic ACCase by oleic acid (18:1) -acyl-carrier protein (ACP), causing reduced fatty acid synthesis, has been described in *Brassica napus* (Andre et al., 2012). Thus, removing SCFA either by increasing the FAE reaction or from the transformation of free FA to TAG can lead to increases in total FA.

**TABLE 1:** Genetic engineering experiments and model predictions.

Enzyme target	Modification	Oil content (OC)	Model prediction	References
Wrinkled1 (glycolytic enzymes)	Downregulation of HK, PFK, PK	No FA accumulation	90% decrease	Focks and Benning, 1998
Plastidial pyruvate kinase	KO	No FA accumulation	92% decrease	Andre and Benning, 2007
ACCase	10–20-fold increase	5% increase	2.1% increase	Roesler et al., 1997
Cell wall invertase	Upregulation	No change	<1% increase	Tomlinson et al., 2004
Hexokinase	Upregulation	No change	1.4% increase	Tomlinson et al., 2004
FA synthase enzymes	Overexpression of FAS complex enzymes	No change or slight decrease in FA	<1% increase	Thelen and Ohlrogge, 2002
PDH kinase	KO – results in two–threefold increase in PDC activity	15% increase in seed oil and weight (no change on weight basis)	1% increase	Zou et al., 1999
Cytosolic ATP citrate lyase*	Overexpression	16% increase in FA content	19% increase	Rangasamy and Ratledge, 2000
Downstream processing of SCFA**	Expression of yeast sn-2 acetyltransferase	8–48% increase in OC (DW basis)	18% increase	Zou et al., 1997
Formation of oil bodies**	Overexpression of GPD1 to increase TAGs	40% increase in OC	23% increase	Vigeolas et al., 2007
Epimerase***	KO	35% increase in TAG at heart stage	18% increase	This study
Cytosolic ACCase***	KO	29% decrease in TAG at heart stage	27% decrease	This study

*This study was performed on leaves of Arabidopsis. **Studies performed on Brassica napus. SCFA, short chain fatty acids. ***Metabolome data obtained from mutants cultivated in our lab (see section “Materials and Methods”).

From the model, we have selected 2 predictions and conducted experimental validation: one for the cytosolic ACCase (ACC, AT1G36160) (Baud et al., 2003) and the other for ribulose-5-phosphate epimerase (EPI, AT5G61410) (Favery et al., 1998). Cytosolic ACCase is responsible for the malonyl-CoA pool necessary for the production of very long chain fatty acids found in TAG and in cuticular waxes (Lü et al., 2011). Also, ribulose-5-phosphate 3-epimerase is a key enzyme in the reductive Calvin cycle and the oxidative pentose phosphate pathway, which play a crucial role in cells by producing NADPH that is required in numerous biosynthetic reactions, including fatty acid synthesis (Favery et al., 1998). Loss of function of either of these two genes results in defective embryo development, confirming their importance to embryo and seed development (Tzafrir et al., 2004). Interestingly, experimental results showed that the *acc* mutant had lower FA and the EPI mutant (*epi*) had higher FA in the heart stage of embryos (Table 1), which are in agreement with the predictions and published reports. These results suggest that the dynamic model can produce reliable predictions of FA content after single gene perturbations. It should be noted that 12 sensitive reactions in Figure 2 do not connect directly to the FA biosynthetic pathway, highlighting the importance of the integration of the pathways and gene expression profiles in network modeling. Although some of the predictions from the model have been experimentally validated, suggesting the usefulness of the model, we like to point out that the current model has certain limitations, because at the present time the exact volume information for the key subcellular compartments of cells of the plant embryo, the cytosol, plastid, and mitochondria, have not been determined and therefore are not available for the *Arabidopsis* embryo developmental stages. Ideally, the model would incorporate the volume information into the differential equations in each compartment to adjust the metabolite concentrations in each compartment. By doing so, we could more accurately model the reactions and make more accurate predictions. This limitation will be addressed in the future when the compartment volumes are available for *Arabidopsis* embryo development.

## Conclusion

Integration of gene expression, enzymes and metabolites in the same conceptual model is critically important for improving our understanding of dynamics and interactions in biological systems (Fendt et al., 2010; Kotte et al., 2010). These integrative approaches are also expected to have major implications in metabolic engineering, drug design and synthetic biology (Schmid and Blank, 2010). Here the metabolic network for storage compound accumulation in *Arabidopsis* seeds was investigated using a mathematical model that integrates gene expression profiles during embryo development. The dynamic model was then used to predict the effects of single gene perturbations on seed FA content. We showed that the predicted results are largely validated either from the literature or from new experiments we conducted. These observations highlight that the dynamic model also generates biological insights for understanding the genetic basis for seed FA content determination. The results suggest that our model can be used to guide metabolic engineering for oil seed crops.

The model can be used to integrate high-throughput data such as gene expression datasets by providing a framework for data analysis and testable predictions. We believe that our modeling framework could be used to further integrate quantitative and time-course metabolomic and proteomic datasets. Thus, the development of such modeling frameworks will be improved by the development of experimental techniques to improve data analysis and experimental design.

Although high-throughput technologies have generated large omics datasets, it is still very challenging to connect and assign ‘causal’ signals from these data to phenotypic traits and diseases. Several factors make it difficult to dissect such ‘causal’ or ‘driving’ signals: the data are very complex, the biological systems are also very complex and many genes in the systems are highly interdependent/interconnected and correlated. Moreover, phenotypic traits and diseases are often controlled by multiple genes. Therefore, most of the current analysis of omic data has been focused on association but not identification of the “causal” signals. This work provides a mathematical framework for dissecting gene networks to identify the key gene “hubs” that play key roles in important plant traits associated with seed oil content, providing an insight for potential application in synthetic biology.

## Data Availability Statement

The original contributions generated for this study are included in the article/Supplementary Material, further inquiries can be directed to the corresponding author/s.

## Author Contributions

EW, MC, DX, and RD developed the study concept. DX and MC performed the experiments and collected the data. MC, EW, DX, PG, and JZ analyzed the data. MC and EW performed the statistical analysis. MC, DX, EW, PG, JZ, LK, and RD interpreted the results and prepared the manuscript, with support from all authors.

## Conflict of Interest

The authors declare that the research was conducted in the absence of any commercial or financial relationships that could be construed as a potential conflict of interest.

## Footnotes

1 www.brenda-enzymes.org

2 www2.bri.nrc.ca/plantembryo

3 www.genome.jp/kegg/

4 www.brenda-enzymes.com

5 www.brenda-enzymes.org

## Supplementary Material

Supplementary Figure 1 Normalized gene expression profiles and simulated enzymes profiles.

Supplementary Figure 2 Pathways of the metabolic model.

Supplementary Figure 3 Metabolic fluxes and concentrations ratios. Lines show model simulations for typical ratios.

Supplementary Table 1 Development stages of samples used in this study.

Supplementary Table 2 List of genes associated to enzymatic reactions of the model.

Supplementary Table 3 Ordinary differential equations (ODEs) for cytosolic metabolism and energy.

Supplementary Table 4 Kinetic equations for central metabolism fluxes.

Supplementary Table 5 Ordinary differential equations for plastidic metabolism and FA.

Supplementary Table 6 Kinetic equations FA synthesis and elongation.

Supplementary Table 7 Ordinary differential equations for mitochondrial metabolism.

Supplementary Table 8 Kinetic equations for mitochondrial metabolism fluxes.

Supplementary Table 9 Ordinary differential equations for growth and storage products accumulation.

Supplementary Table 10 Kinetic equations for transport and storage fluxes.

Supplementary Table 11 Kinetic equations for FA oxidation and maintenance.

Supplementary Table 12 Parameters of the model.
