# Supplementary material for: Integrative Modeling of Gene Expression and Metabolic Networks of Arabidopsis Embryos for Identification of Seed Oil Causal Genes
Source: Front Plant Sci. 2021 Apr 6;12:642938. doi: 10.3389/fpls.2021.642938 (PMC8056077; doi:10.3389/fpls.2021.642938)
Supplement: Supplementary Figure 1 — Normalized gene expression profiles and simulated enzymes profiles. [file Data_Sheet_1.PDF]

Supplementary Tables

Supplementary Table 1: Development stages of samples used in this study.

Development stage	Description	Time (DAF)
Z	Zygote	0-1
Q	Quadrant	2
G	Globular	4
H	Heart	6
T	Torpedo	7
B	Bent	8
M	Mature	12-15

Supplementary Table 2: List of genes associated to enzymatic reactions of the model

Enzyme	Genes	Turnover	Enzyme	Genes	Turnover
RIB**	At1g26910 …	---	PKp	At1g32440	- a
				At5g52920	
GLCT	At5g61520	-	PDHp	At1g01090	-
	At3g19930			At1g30120	
	At1g34580	- a		At2g34590	- a
	At5g26340			At1g34430	
	At4g02050			At3g16950	
CWI	At5G63840	a	ACCp	At5g16390	a
SUCT	At1g22710	-	FAS	At1g74960	
	At2g02860	a			a
	At1g71890	-			
	At5g43610				
PEPT	At5g46110	- a	FASII	At1g08510	- a
PYRT	At3g01550			At3g25110	
AAT	At1g17290	a	FAE	At4g34520	f
GDH	At5g07440	- a	TAG	At2g19450	- a
	At1g51720			At4g25140	
HK	At1g47840	-	ACL	At1g09430	-
	At2g19860	f		At1g10670	a
	At4g29130				
PFK	At2g22480	-	ACC	At1g36160	-
	At4g26270			At2g38040	
	At4g29220-			At5g15530	-
	At4g32840	f		At5g35360	a
	At5g56630	-			
	At5g61580				
PGK	At1g56190-		ME	At1g79750	
	At1g79550	a			a
	At3g12780				

NPG	At2g24270		a	PEPC	At1g68750	-	a
					At2g42600		
PK	At3g49160	-		STAS	At5g19220	-	
	At3g52990		a		At5g48300		f
	At5g08570	-			At5g65685	-	
	At5g63680				At1g11720		
PDH	At1g01090	-		STAD	At4g25000	-	
	At1g24180				At3g29320		
	At1g59900	-	a		At3G46970		f
	At1g30120						
	At2g34590	-					
	At5g50850						
CS	At2g42790	-		SPRT	At4g28520	-	
	At2g44350				At5g44120		
	At3g58740	-			At1g03880	-	
	At3g58750		a		At1g03890		a
	At3g60100				At4g27150	-	
					At4g27160		
					At5g54740		
ICDH	At1g54340	-		OP***	At2g31040	-	
	At1g65930				At1g51650		
	At5g14590	-			At5g47030	-	
	At1g32480				At5g08670		
	At2g17130	-	a		At5g08680	-	a
	At3g09810				At5g08690		
	At4g35260	-			At2g33040	-	
	At4g35650				At2g07698		
	At5g03290				At5g13450		
KDH	At5g65750			SUS	At5g20830	-	
			a		At5g49190		s
					At4g02280		

SDH	At3g27380	-		EPI	At1g63290	-	
	At5g40650		a		At3g01850		a
	At5g65165				At5g61410		
MDH	At1g53240	-		FAOX	At3g14360	-	
	At3g15020		a		At5g38520		a
					At4g16070	-	
					At2g42690		
RUB	At1g67090	-		GPDH	At1g24280	-	
	At5g38410				At5g13110		
	At5g38420	-			At5g35790	-	
	At5g38430		s		At5g40760		f
					At3g02360	-	
					At5g41670		
				ICL	At3g21720	-	a
					At5g03860		

*f = fast, a = average, s = slow, as defined previously.

**Genes for RIB (≈200) were taken from reference [3].

***The oxidative phosphorylation for ATP production is a complex system involving many genes. Here, we limit our use of expression data to the genes for mitochondrial ATPases that produces ATP from the mitochondrial membrane potential.

Supplementary Table 3: ODEs for cytosolic metabolism and energy

Variables		Differential equations*
HEXe	Extracellular hexoses	$\frac{dHEXe}{dt} = 2 \cdot v_{cwi} - v_{glct}$
HEX	Hexoses	$\frac{dHEX}{dt} = v_{glct} - v_{hk} - v_{sus} - \mu \cdot HEX$
HEXP	Hexoses phosphate	$\frac{dHEXP}{dt} = v_{hk} - v_{pfk} - v_{gpdh} + 2 \cdot v_{epi} - v_{stas} + v_{stad} - v_{sus} - v_{cws} - \mu \cdot HEXP$
R5P	Ribulose-5-phosphate	$\frac{dR5P}{dt} = v_{gpdh} - 3 \cdot v_{epi} - v_{rub} - \mu \cdot R5P$
GAP	Glyceraldehyde-3-phosphate	$\frac{dGAP}{dt} = 2 \cdot v_{pfk} + v_{epi} - v_{pgk} - v_{npg} - v_{tag} + v_{faox} - \mu \cdot GAP$
PEP	Phosphoenol-pyruvate	$\frac{dPEP}{dt} = v_{pgk} + v_{npg} - v_{pk} - v_{pepT} - v_{pepc} - \mu \cdot PEP$
PYR	Pyruvate	$\frac{dPYR}{dt} = v_{pk} - v_{pdh} + v_{aat} - v_{pyrT} - v_{sprt} - \mu \cdot PYR$
ACoA**	Acetyl-CoA	$\frac{dACoA}{dt} = v_{acl} - v_{acc} - \mu \cdot ACoA$
MCoA**	Malonyl-CoA	$\frac{dMCoA}{dt} = v_{acc} - v_{fae} - \mu \cdot MCoA$
NADPH***	Nicotinamide adenine dinucleotide phosphate	$\frac{dNADPH}{dt} = 2 \cdot v_{gpdh} + v_{npg} + v_{me} - 14 \cdot v_{fas} - v_{fas2} - 2 \cdot v_{fae} + v_{ox}$
ATP****	Adenosine triphosphate	$\frac{dATP}{dt} = -v_{hk} - v_{pfk} + v_{pk} + v_{pgk} + v_{pkp} + 3 \cdot v_{op} - v_{atpase} - 8 \cdot v_{fas} - v_{fas2} - v_{fae} - 5 \cdot v_{sprt} - v_{acl} - v_{acc} - v_{accp} - v_{cws}$

*For better readability, $v_{fae} = v_{fae16} + v_{fae18} + v_{fae20}$; $v_{tag} = v_{tag16} + v_{tag18} + v_{tag20} + v_{tag22}$ and $v_{faox} = v_{faox16} + v_{faox18} + v_{faox20} + v_{faox22}$

**The conserved moiety: $CoA = CoA_{TOT} - ACoA - MCoA$ is associated with these variables.

***The conserved moiety: NADP = $NADPH_{TOT}$ - NADPH is associated with this variable.

****The conserved moiety: ADP = ATP_{TOT} - ATP is associated with this variable.

Supplementary Table 4: Kinetic equations for central metabolism fluxes

Enzyme		Kinetic equation
HK	Hexokinase	$v_{hk} = E_{hk} \cdot k_{hk} \cdot \frac{HEX}{K_{M,HEX} + HEX} \cdot \frac{ATP}{K_{M,ATP} + ATP} \cdot \frac{1}{1 + (HEXP/K_{I,HEXP})^4}$
PFK	Phosphofructokinase	$v_{pfk} = E_{pfk} \cdot k_{pfk} \cdot \frac{HEXP}{K_{M,HEXP} + HEXP} \cdot \frac{ATP}{K_{M,ATP} + ATP} \cdot \frac{1}{1 + (ATP/K_{I,ATP})^4}$
PGK	Phosphoglycerate kinase	$v_{pgk} = E_{pgk} \cdot \left[kf_{pgk} \cdot \frac{GAP}{K_{M,GAP} + GAP} \cdot \frac{ADP}{K_{M,ADP} + ADP} \cdot \frac{NAD}{K_{M,NAD} + NAD} - kr_{pgk} \cdot PEP \cdot ATP \cdot NADH \right]$
NPG	Non phosphorylating glycerate kinase	$v_{npg} = E_{npg} \cdot k_{npg} \cdot \frac{GAP}{K_{M,GAP} + GAP} \cdot \frac{NADP}{K_{M,NADP} + NADP} \cdot \frac{1}{1 + (PEP/K_{I,PEP})^4}$
PK	Pyruvate kinase	$v_{pk} = E_{pk} \cdot k_{pk} \cdot \frac{PEP}{K_{M,PEP} + PEP} \cdot \frac{ADP}{K_{M,ADP} + ADP} \cdot \frac{1}{1 + (ATP/K_{I,ATP})^4} \cdot \frac{1}{1 + (PYR/K_{I,PYR})^6}$
GPDH	G6P dehydrogenase	$v_{gpdh} = E_{gpdh} \cdot k_{gpdh} \cdot \frac{HEXP}{K_{M,HEXP} + HEXP} \cdot \frac{NADP}{K_{M,NADP} + NADP} \cdot \frac{1}{1 + (NADPH/K_{I,NADPH})^4}$
EPI	Epimerase	$v_{epi} = E_{epi} \cdot k_{epi} \cdot \frac{R5P}{K_{M,R5P} + R5P}$
PEPC	PEP carboxylase	$v_{pepc} = E_{pepc} \cdot k_{pepc} \cdot \frac{PEP}{K_{M,PEP} + PEP} \cdot \frac{1}{1 + (OAA/K_{I,OAA})^4}$

Supplementary Table 5: ODEs for plastidic metabolism and FA

Variable		Differential equation
PEPp	Plastidic PEP	$\frac{dPEPp}{dt} = 2 \cdot v_{rub} + v_{pepT} - v_{pkp} - \mu \cdot PEPp$
PYRp	Plastidic PYR	$\frac{dPYRp}{dt} = v_{pkp} + v_{pyrT} - v_{pdhp} - \mu \cdot PYRp$
ACoAp*	Plastidic ACoA	$\frac{dACoAp}{dt} = v_{pdhp} - v_{accp} - v_{fas1} - \mu \cdot ACoAp$
MCoAp *	Plastidic MCoA	$\frac{dMCoAp}{dt} = v_{accp} - 7 \cdot v_{fas1} - v_{fas2} - \mu \cdot MCoAp$
FA16	16C FA	$\frac{dFA16}{dt} = v_{fas1} - v_{fas2} - v_{fae16} - 3 \cdot v_{tag16} - \mu \cdot FA16$
FA18	18C FA	$\frac{dFA18}{dt} = v_{fas2} + v_{fae16} - v_{fae18} - 3 \cdot v_{tag18} - \mu \cdot FA18$
FA20	20C FA	$\frac{dFA20}{dt} = v_{fae18} - v_{fae20} - 3 \cdot v_{tag20} - \mu \cdot FA20$
FA22	22C FA	$\frac{dFA22}{dt} = v_{fae20} - 3 \cdot v_{tag22} - \mu \cdot FA22$
TAG16	16C FA in TAG	$\frac{dTAG16}{dt} = v_{tag16} - v_{faox16} - \mu \cdot TAG16$
TAG18	18C FA in TAG	$\frac{dTAG18}{dt} = v_{tag18} - v_{faox18} - \mu \cdot TAG18$
TAG20	20C FA in TAG	$\frac{dTAG20}{dt} = v_{tag20} - v_{faox20} - \mu \cdot TAG20$
TAG22	22C FA in TAG	$\frac{dTAG22}{dt} = v_{tag22} - v_{faox22} - \mu \cdot TAG22$

*The conserved moiety: CoAp = $CoAp_{TOT}$ - ACoAp - MCoAp is associated with these variables.

Supplementary Table 6: Kinetic equations FA synthesis and elongation

**Enzyme**		**Kinetic equation**
RUB	Rubisco	$v_{rub} = E_{rub} \cdot k_{rub} \cdot \frac{R5P}{K_{M,R5P} + R5P} \cdot \frac{1}{1+\left(PEP/K_{I,PEP}\right)^6}$
PKp	Plastidial pyruvate kinase	$v_{pkp} = E_{pkp} \cdot k_{pkp} \cdot \frac{PEP}{K_{M,PEP} + PEP} \cdot \frac{ADP}{K_{M,ADP} + ADP} \cdot \frac{1}{1+\left(ATP/K_{I,ATP}\right)^4} \cdot \frac{1}{1+\left(PYR/K_{I,PYR}\right)^6}$
PDHp	Plastidial pyruvate dehydrogenase	$v_{pdhp} = E_{pdhp} \cdot k_{pdhp} \cdot \frac{PYRp}{K_{M,PYR} + PYRp} \cdot \frac{CoAp}{K_{M,CoA} + CoAp}$
ACCp	Plastidial ACoA carboxylase	$v_{accp} = E_{accp} \cdot k_{accp} \cdot \frac{ACoAp}{K_{M,ACoAp} + ACoAp} \cdot \frac{ATP}{K_{M,ATP} + ATP} \cdot \frac{1}{1+\left(MCoAp/K_{I,MCoAp}\right)^4}$
ACC	Cytosolic ACoA carboxylase	$v_{acc} = E_{acc} \cdot k_{acc} \cdot \frac{ACoA}{K_{M,ACoA} + ACoA} \cdot \frac{ATP}{K_{M,ATP} + ATP} \cdot \frac{1}{1+\left(MCoA/K_{I,MCoA}\right)^4}$
ACL	ATP citrate lyase	$v_{acl} = E_{acl} \cdot k_{acl} \cdot \frac{CIT}{K_{M,CIT} + CIT} \cdot \frac{ATP}{K_{M,ATP} + ATP} \cdot \frac{CoA}{K_{M,CoA} + CoA} \cdot \frac{1}{1+\left(OAA/K_{I,OAA}\right)^4}$
FAS	FA synthase	$v_{fas} = E_{fas} \cdot k_{fas} \cdot \frac{ACoAp}{K_{M,ACoA} + ACoAp} \cdot \frac{MCoAp}{K_{M,MCoA} + MCoAp} \cdot \frac{ATP}{K_{M,ATP} + ATP} \cdot \frac{NADPH}{K_{M,NADPH} + NADPH} \cdot \frac{1}{1+\left((FA16+FA18)/K_{I,FA}\right)^6}$
FAS2	FA synthase II	$v_{fasII} = E_{fasII} \cdot k_{fasII} \cdot \frac{FA16}{K_{M,FA16} + FA16} \cdot \frac{MCoAp}{K_{M,MCoA} + MCoAp} \cdot \frac{ATP}{K_{M,ATP} + ATP} \cdot \frac{NADPH}{K_{M,NADPH} + NADPH} \cdot \frac{1}{1+\left(FA18/K_{I,FA}\right)^6}$
FAE16	FA elongase (16→18)	$v_{fae16} = E_{fae} \cdot k_{fae16} \cdot FA16 \cdot \frac{MCoA}{K_{M,MCoA} + MCoA} \cdot \frac{ATP}{K_{M,ATP} + ATP} \cdot \frac{NADPH}{K_{M,NADPH} + NADPH}$

FAE18	FA elongase (18→20)	$v_{fae18} = E_{fae} \cdot k_{fae18} \cdot FA18 \cdot \frac{MCoA}{K_{M,MCoA} + MCoA} \cdot \frac{ATP}{K_{M,ATP} + ATP} \cdot \frac{NADPH}{K_{M,NADPH} + NADPH}$
FAE20	FA elongase (20→22)	$v_{fae20} = E_{fae} \cdot k_{fae20} \cdot FA20 \cdot \frac{MCoA}{K_{M,MCoA} + MCoA} \cdot \frac{ATP}{K_{M,ATP} + ATP} \cdot \frac{NADPH}{K_{M,NADPH} + NADPH}$

Supplementary Table 7: ODEs for mitochondrial metabolism

**Variable**		**Differential equation**
ACoAm*	Mitochondrial ACoA	$\frac{dACoAm}{dt} = v_{pdh} - v_{cs} - v_{icl} + v_{faox} - \mu \cdot ACoAm$
CITm	Citrate	$\frac{dCITm}{dt} = v_{cs} - v_{icdh} - v_{acl} - v_{icl} - \mu \cdot CITm$
KGm	α-ketoglutarate	$\frac{dKGm}{dt} = v_{icdh} - v_{kdh} - v_{gdh} - \mu \cdot KGm$
SUCCm	Succinate	$\frac{dSUCCm}{dt} = v_{kdh} - v_{sdh} + v_{icl} - v_{ora} - \mu \cdot SUCCm$
MALm	Malate	$\frac{dMALm}{dt} = v_{sdh} - v_{mdh} + v_{icl} - v_{me} - \mu \cdot MALm$
OAAm	Oxaloacetate	$\frac{dOAAm}{dt} = v_{mdh} - v_{cs} + v_{pepc} + v_{acl} - \mu \cdot OAAm$
NADH**	Nicotinamide adenine nucleotide	$\frac{dNADH}{dt} = v_{pgk} + v_{pdc} + v_{icdh} + v_{kdh} + v_{mdh} - v_{op} - v_{leak}$

*For better clarity, $v_{faox} = 24 \cdot v_{faox16} + 27 \cdot v_{faox18} + 30 \cdot v_{faox20} + 33 \cdot v_{faox22}$; where the stoichiometric coefficients account for the fact that TAG, with 3 x (16-22C) are degraded into ACoA (2C). The conserved moiety CoAm = $CoAm_{TOT}$ - ACoAm is associated with this variable.

**The conserved moiety NAD = $NADH_{TOT}$ – NADH is associated with this variable.

Supplementary Table 8: Kinetic equations for mitochondrial metabolism fluxes

Enzyme		Kinetic equation
PDH	Mitochondrial pyruvate dehydrogenase	$v_{pdh} = E_{pdh} \cdot k_{pdh} \cdot \frac{PYR}{K_{M,PYR} + PYR} \cdot \frac{CoA}{K_{M,CoA} + CoA}$
CS	Citrate synthase	$v_{cs} = E_{cs} \cdot k_{cs} \cdot \frac{ACoA}{K_{M,ACoA} + ACoA} \cdot \frac{OAA}{K_{M,OAA} + OAA} \cdot \frac{1}{1+\left(ATP/K_{I,ATP}\right)^4} \cdot \frac{1}{1+\left(CIT/K_{I,CIT}\right)^4}$
ICDH	Isocitrate dehydrogenase	$v_{icdh} = E_{icdh} \cdot k_{icdh} \cdot \left[kf_{icdh} \cdot CIT \cdot NAD - kr_{icdh} \cdot KG \cdot NADH\right]$
KDH	α-ketoglutarate dehydrogenase	$v_{kdh} = E_{kdh} \cdot k_{kdh} \cdot \frac{KG}{K_{M,KG} + KG} \cdot NAD$
SDH	Succinate dehydrogenase	$v_{sdh} = E_{sdh} \cdot k_{sdh} \cdot SUCC$
MDH	Malate dehydrogenase	$v_{mdh} = E_{mdh} \cdot k_{mdh} \cdot \frac{MAL}{K_{M,MAL} + MAL} \cdot NAD \cdot \frac{1}{1+\left(OAA/K_{I,OAA}\right)^4}$
ME	Malic enzyme	$v_{me} = E_{me} \cdot k_{me} \cdot \frac{MAL}{K_{M,MAL} + MAL} \cdot \frac{NADP}{K_{M,NADP} + NADP} \cdot \frac{1}{1+\left(PYR/K_{I,PYR}\right)^6}$
OP	Oxidative phosphorylation (ATP synthase)	$v_{op} = E_{op} \cdot k_{op} \cdot \frac{NADH}{K_{M,NADH} + NADH} \cdot \frac{ADP}{K_{M,ADP} + ADP} \cdot \frac{1}{1+10 \cdot ATP/ADP}$

Supplementary Table 9: ODEs for growth and storage products accumulation

Variable		Differential equation
STA	Starch	$\frac{dSTA}{dt} = v_{stas} - v_{stad} - \mu \cdot STA$
SUC	Sucrose	$\frac{dSUC}{dt} = v_{sucT} + v_{sus} - \mu \cdot SUC$
SPRT	Storage proteins	$\frac{dSPRT}{dt} = v_{sprt} - \mu \cdot SPRT$
ORA	Storage organic acids	$\frac{dORA}{dt} = v_{ora} - \mu \cdot ORA$
X	Embryo mass	$\frac{dX}{dt} = \mu \cdot X$ with: $\mu = \mu_{\max} \cdot \left(1 - \frac{X}{X_{\max}}\right)$

Supplementary Table 10: Kinetic equations for transport and storage fluxes

Enzyme		Kinetic equation
CWI	Cell wall invertase	$v_{cwi} = E_{cwi} \cdot k_{cwi} \cdot \frac{SUCe}{K_{M,SUCe} + SUCe}$
GLCT	Glucose transport (endosp.→ cytosol)	$v_{glcT} = E_{glcT} \cdot k_{glcT} \cdot \left[\frac{GLCe}{K_{M,GLCT} + GLCe} - \frac{GLC}{K_{M,GLCT} + GLC}\right] \cdot \frac{1}{1 + \left(SUC/K_{I,SUC}\right)^{\text{[illegible]}}}$
SUCT	Sucrose transport (endosp.→ cytosol)	$v_{sucT} = E_{sucT} \cdot k_{sucT} \cdot \left[\frac{SUCe}{K_{M,SUCT} + SUCe} - \frac{SUC}{K_{M,SUCT} + SUC}\right]$
PEPT	PEP transport (cytosol→plastids)	$v_{pepT} = E_{pepT} \cdot k_{pepT} \cdot PEP \cdot \frac{1}{1 + \left(PEPp/K_{I,PEP}\right)^4}$
PYRT	PYR transport (cytosol→plastids)	$v_{pyrT} = E_{pyrT} \cdot k_{pyrT} \cdot PYR \cdot \frac{1}{1 + \left(PYRp/K_{I,PYR}\right)^4}$
GDH	Glutamate dehydrogenase	$v_{gdh} = E_{gdh} \cdot \left[kf_{gdh} \cdot KG \cdot NADH - kr_{gdh} \cdot GLU \cdot NAD\right]$
AAT	Alanine aminotransferase	$v_{aat} = E_{aat} \cdot k_{aat} \cdot \left[ALA - PYR\right]$
SUS	Sucrose synthase	$v_{sus} = E_{sus} \cdot \left[\frac{kf_{sus} \cdot HEX \cdot HEXP - kr_{sus} \cdot SUC}{1 + HEX \cdot HEXP/K_{M,HEX} + SUC/K_{M,SUC}}\right] \cdot \frac{1}{1 + \left(SUC/K_{I,SUC}\right)^4}$
STAS	Starch synthesis	$v_{stas} = E_{stas} \cdot k_{stas} \cdot \frac{HEXP}{K_{M,HEXP} + HEXP} \cdot \frac{1}{1 + \left(STA/K_{I,STA}\right)^4}$
STAD	Starch degradation	$v_{stad} = E_{stad} \cdot k_{stad} \cdot \frac{STA}{K_{M,STA} + STA} \cdot \frac{1}{1 + \left(HEXP/K_{I,HEXP}\right)^4}$
SPRT	Storage protein synthesis	$v_{sprt} = mRNA_{sprt} \cdot RIB \cdot k_{sprt} \cdot \frac{PYR}{K_{M,PYR} + PYR} \cdot \frac{ATP}{K_{M,ATP} + ATP} \cdot \frac{1}{1 + \left(SPRT/K_{I,SPRT}\right)^4}$

CWS	Cell wall synthesis	$v_{cws} = k_{cws} \cdot \mu \cdot \frac{HEXP}{K_{M,HEXP} + HEXP} \cdot \frac{ATP}{K_{M,ATP} + ATP}$
ORA	Organic acids accumulation	$v_{ora} = kf_{ora} \cdot \frac{SUCC}{K_{M,SUCC} + SUCC} - kr_{ora} \cdot ORA$
TAG16	TAG formation (FA16→TAG16)	$v_{tag16} = E_{tag} \cdot k_{tag16} \cdot FA16 \cdot \frac{GAP}{K_{M,GAP} + GAP}$
TAG18	TAG formation (FA18→TAG18)	$v_{tag18} = E_{tag} \cdot k_{tag18} \cdot FA18 \cdot \frac{GAP}{K_{M,GAP} + GAP}$
TAG20	TAG formation (FA20→TAG20)	$v_{tag20} = E_{tag} \cdot k_{tag20} \cdot FA20 \cdot \frac{GAP}{K_{M,GAP} + GAP}$
TAG22	TAG formation (FA22→TAG22)	$v_{tag22} = E_{tag} \cdot k_{tag22} \cdot FA22 \cdot \frac{GAP}{K_{M,GAP} + GAP}$

Supplementary Table 11: Kinetic equations for FA oxidation and maintenance

**Enzyme**		**Kinetic equation**
FAOX16	FA oxidation (TAG16 →ACoA)	$v_{faox16} = E_{faox} \cdot k_{faox} \cdot TAG16 \cdot \frac{CoA}{K_{M,CoA} + CoA}$
FAOX18	FA oxidation (TAG18 →ACoA)	$v_{faox18} = E_{faox} \cdot k_{faox} \cdot TAG18 \cdot \frac{CoA}{K_{M,CoA} + CoA}$
FAOX20	FA oxidation (TAG20 →ACoA)	$v_{faox20} = E_{faox} \cdot k_{faox} \cdot TAG20 \cdot \frac{CoA}{K_{M,CoA} + CoA}$
FAOX22	FA oxidation (TAG22 →ACoA)	$v_{faox22} = E_{faox} \cdot k_{faox} \cdot TAG22 \cdot \frac{CoA}{K_{M,CoA} + CoA}$
ICL	Isocitrate lyase (Glyoxylate cycle)	$v_{icl} = E_{icl} \cdot k_{icl} \cdot \frac{ACoAm}{K_{M,ACoA} + ACoAm} \cdot \frac{CIT}{K_{M,CIT} + CIT} \cdot \frac{1}{1 + \left(ATP/K_{I,ATP}\right)^4} \cdot \frac{1}{1 + \left(SUCC/K_{I,SUCC}\right)^6}$
leak	NADH consumption to maintain mito. membrane potential	$v_{leak} = k_{leak} \cdot \frac{NADH}{K_{M,NADH} + NADH}$
OX	NADPH consumption by endogenous oxidative stress	$v_{ox} = k_{ox} \cdot \frac{NADPH}{K_{M,NADPH} + NADPH}$
ATPase	ATP consumption for growth and maintenance	$v_{atpase} = (\alpha \cdot \mu + \beta) \cdot k_{atpase} \cdot \frac{ATP}{K_{M,ATP} + ATP}$

Supplementary Table 12 Parameters of the model

Parameter name	Value	Description	Units
k_{cwi}	147.4	CWI reaction constant	$\mu M \cdot h^{-1}$
k_{glcT}	196	GLC transporter reaction constant	$\mu M \cdot h^{-1}$
k_{sucT}	0.5	SUC transporter reaction constant	$\mu M \cdot h^{-1}$
k_{pepT}	0.862	PEP transporter reaction constant	$\mu M \cdot h^{-1}$
k_{pyrT}	0.862	PYR transporter reaction constant	$\mu M \cdot h^{-1}$
kf_{sus}	0.05	SUS forward reaction constant	$\mu M \cdot h^{-1}$
kr_{sus}	0.01	SUS reverse reaction constant	$\mu M \cdot h^{-1}$
k_{ATPase}	58.7	ATPase reaction constant	$\mu M \cdot h^{-1}$
k_{stas}	30	Reaction constant for STA synthesis	$\mu M \cdot h^{-1}$
k_{stad}	75.8	Reaction constant for STA degradation	$\mu M \cdot h^{-1}$
k_{hk}	815	HK reaction constant	$\mu M \cdot h^{-1}$
k_{pfk}	1175	PFK reaction constant	$\mu M \cdot h^{-1}$
kf_{pgk}	75.4	PGK forward reaction constant	$\mu M \cdot h^{-1}$
kr_{pgk}	1.05	PGK reverse reaction constant	$\mu M^{-2} \cdot h^{-1}$
k_{npg}	126.2	NPG reaction constant	$\mu M \cdot h^{-1}$
k_{pk}	33	PPK reaction constant	$\mu M \cdot h^{-1}$
k_{pepc}	23.4	PEPC reaction constant	$\mu M \cdot h^{-1}$
k_{pdh}	251	PDH reaction constant	$\mu M \cdot h^{-1}$
k_{cs}	35	CS reaction constant	$\mu M \cdot h^{-1}$
kf_{icdh}	6.89	ICDH forward reaction constant	$\mu M \cdot h^{-1}$
kr_{icdh}	5	ICDH reverse reaction constant	$\mu M \cdot h^{-1}$
k_{kdh}	33	KDH reaction constant	$\mu M \cdot h^{-1}$
k_{sdh}	20.6	SDH reaction constant	$\mu M \cdot h^{-1}$
k_{mdh}	1.02	MDH reaction constant	$\mu M \cdot h^{-1}$
k_{op}	605	Oxidative phosphorylation reaction constant	$\mu M \cdot h^{-1}$
k_{me}	1.8	ME reaction constant	$\mu M \cdot h^{-1}$
k_{acl}	6.8	ACL reaction constant	$\mu M \cdot h^{-1}$

k_{acc}	558	ACC reaction constant	$\mu M \cdot h^{-1}$
k_{aat}	0.75	AAT reaction constant	$\mu M \cdot h^{-1}$
k_{gpdh}	209	G6PDH reaction constant	$\mu M \cdot h^{-1}$
k_{epi}	52.3	EPI reaction constant	$\mu M \cdot h^{-1}$
k_{rub}	192	RUB reaction constant	$\mu M \cdot h^{-1}$
k_{pkp}	79.9	PKp reaction constant	$\mu M \cdot h^{-1}$
k_{pdhp}	123.5	PDHp reaction constant	$\mu M \cdot h^{-1}$
k_{accp}	2500	ACCp reaction constant	$\mu M \cdot h^{-1}$
kf_{gdh}	1.15	GDH forward reaction constant	$\mu M \cdot h^{-1}$
kr_{gdh}	1	GDH reverse reaction constant	$\mu M \cdot h^{-1}$

Supplementary Table 12 Parameters of the model (…continued)

Parameter name	Value	Description	Units
k_{fas}	2504	FA synthase reaction constant	$\mu M \cdot h^{-1}$
k_{fasII}	11.6	FA synthase II reaction constant	$\mu M \cdot h^{-1}$
k_{fae16}	0.125	FA_{16} elongation reaction constant	$\mu M \cdot h^{-1}$
k_{fae18}	0.008	FA_{18} elongation reaction constant	$\mu M \cdot h^{-1}$
k_{fae20}	0.008	FA_{20} elongation reaction constant	$\mu M \cdot h^{-1}$
k_{tag16}	$5 \cdot 10^{-4}$	FA_{16} → TAG reaction constant	$\mu M \cdot h^{-1}$
k_{tag18}	$5 \cdot 10^{-4}$	FA_{18} → TAG reaction constant	$\mu M \cdot h^{-1}$
k_{tag20}	$3.5 \cdot 10^{-3}$	FA_{20} → TAG reaction constant	$\mu M \cdot h^{-1}$
k_{tag22}	$3.5 \cdot 10^{-3}$	FA_{22} → TAG reaction constant	$\mu M \cdot h^{-1}$
k_{faox}	$1.6 \cdot 10^{-3}$	FA oxidation reaction constant	$\mu M \cdot h^{-1}$
k_{icl}	0.95	Isocitrate lyase reaction constant	$\mu M \cdot h^{-1}$
kf_{ora}	0.6	Organic acids storage forward reaction constant	$\mu M \cdot h^{-1}$
kr_{ora}	0.002	Organic acids storage reverse reaction constant	$\mu M \cdot h^{-1}$
k_{cws}	1000	Cell wall synthesis reaction constant	$\mu M \cdot h^{-1}$
k_{leak}	35	Mitochondrial proton leak reaction constant	$\mu M \cdot h^{-1}$
k_{ox}	6.89	Endogenous oxidative stress reaction constant	$\mu M \cdot h^{-1}$
$ATPtot$	2.51	Total amount of ATP + ADP	$\mu M \cdot gDW^{-1}$
$K_{M,GLCT}$	60	Affinity constant of GLC transporter for GLC	$\mu M \cdot gDW^{-1}$
$K_{M,SUCT}$	250	Affinity constant of SUC transporter for SUC	$\mu M \cdot gDW^{-1}$
$K_{M,HEX,hk}$	261	Affinity constant of GLC transporter for GLC	$\mu M \cdot gDW^{-1}$
$K_{M,ATP,hk}$	0.23	Affinity constant of GLC transporter for GLC	$\mu M \cdot gDW^{-1}$
$K_{I,G6P}$	10	Affinity constant of GLC transporter for GLC	$\mu M \cdot gDW^{-1}$
$K_{M,HEXP,pfk}$	1	Affinity constant of GLC transporter for GLC	$\mu M \cdot gDW^{-1}$
k_{sprt}	2.05	Storage protein reaction constant	$\mu M \cdot h^{-1}$

Supplementary Figures

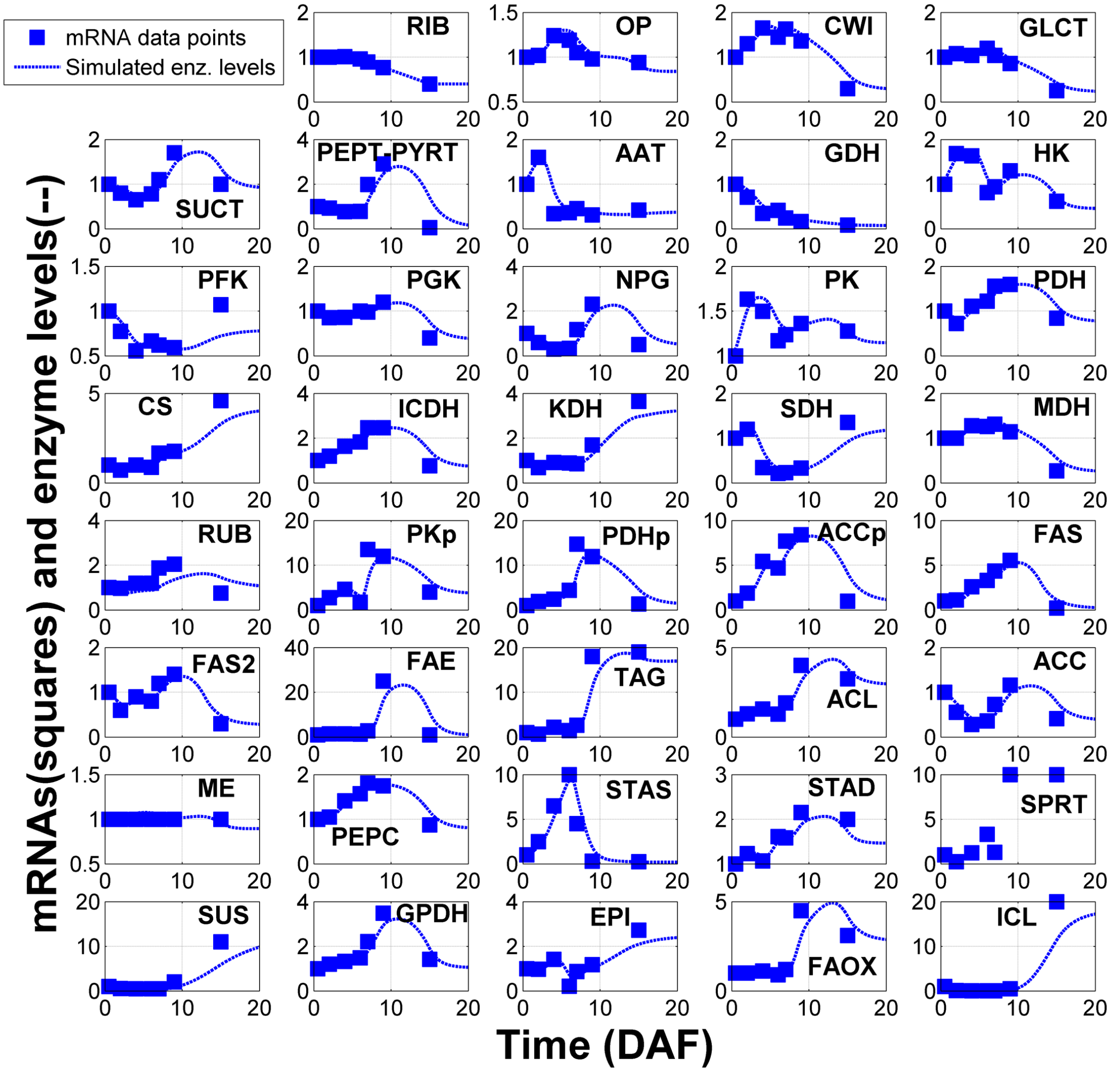

Supplementary Figure 1. Normalized gene expression profiles and simulated enzymes profiles. Squares represent data points from our gene expression database, normalized to an initial value of 1, as described in the text. Dotted lines represent the simulated enzymes profiles. Note that dotted lines will not automatically follow the data, mostly because of enzyme dynamics and changes in RIB as defined in equation S1.

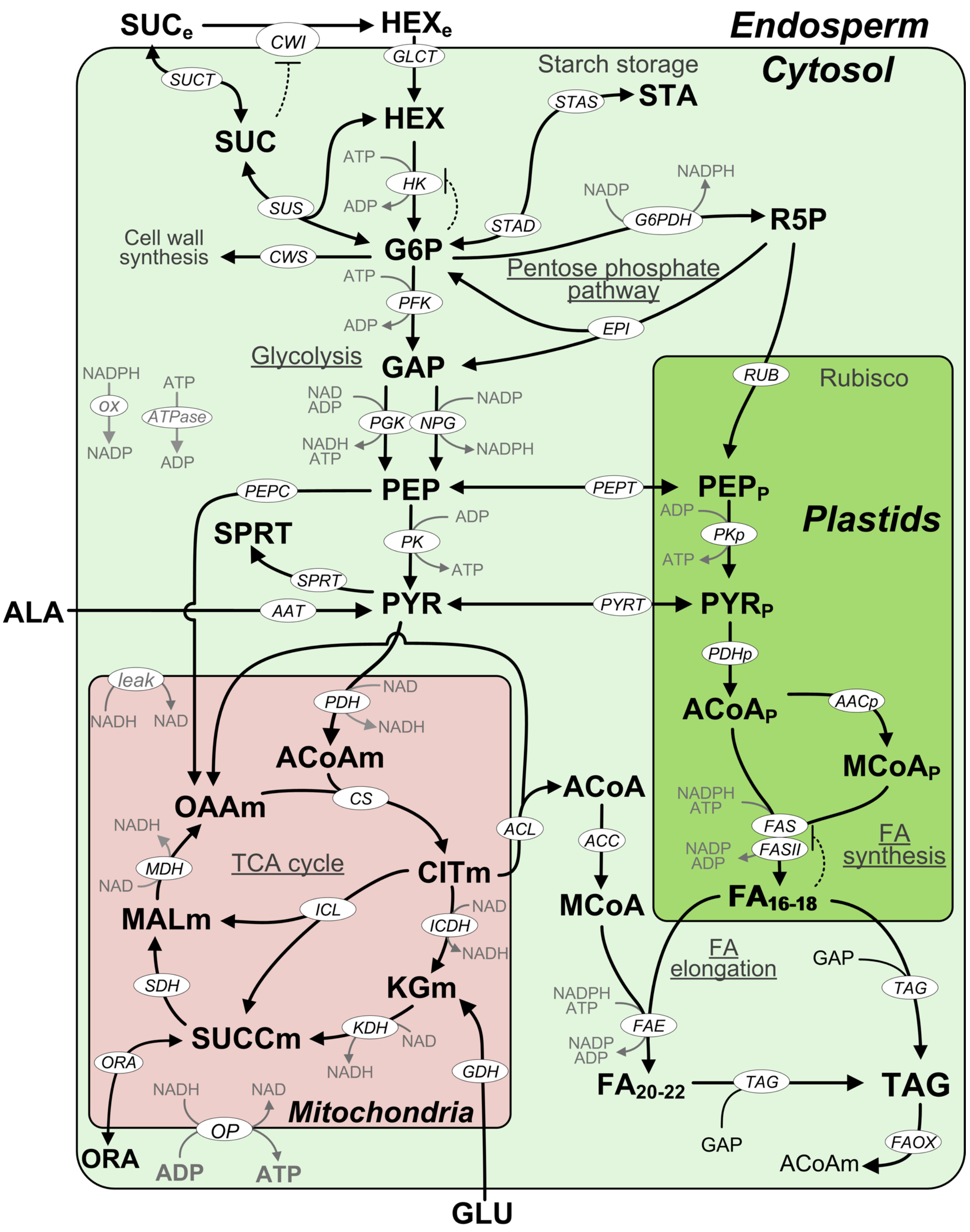

Supplementary Figure 2. Pathways of the metabolic model. Substrates, metabolites and end products are in bold font, cofactors are in grey font and enzymes and reactions are in white ellipses. Full names of enzymes and metabolites are provided in the Supplementary Table 3-12.

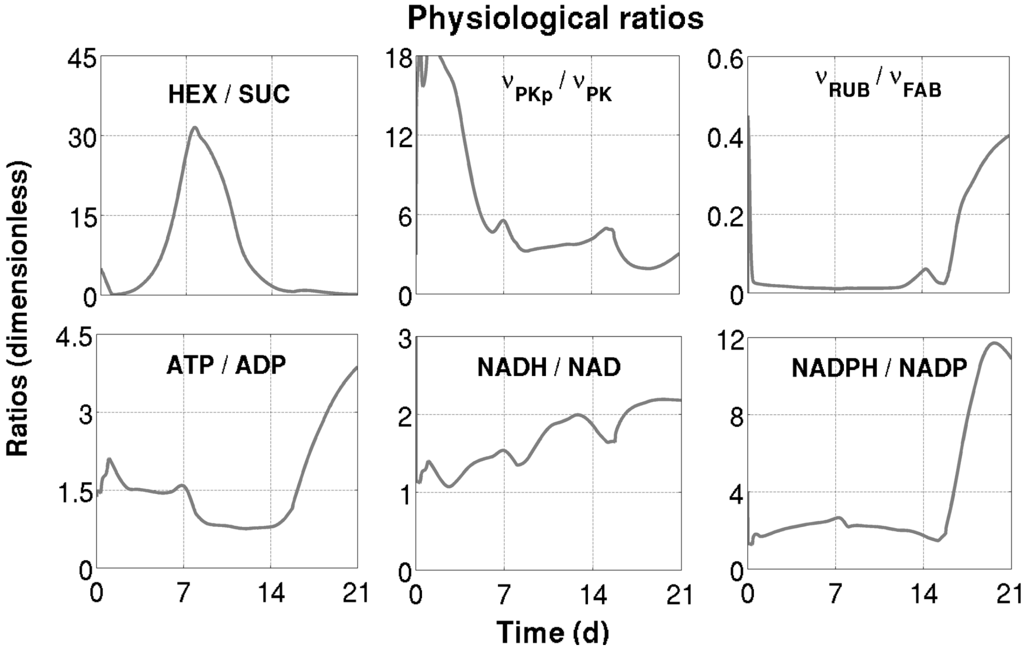

Supplementary Figure 3. Metabolic fluxes and concentrations ratios. Lines show model simulations for typical ratios.
